# Survival Outcomes of Radical Prostatectomy + Extended Pelvic Lymph Node Dissection and Radiotherapy in Prostate Cancer Patients With a Risk of Lymph Node Invasion Over 5%: A Population-Based Analysis

**DOI:** 10.3389/fonc.2020.607576

**Published:** 2020-11-26

**Authors:** Junru Chen, Yuchao Ni, Guangxi Sun, Sha Zhu, Jinge Zhao, Zhipeng Wang, Haoran Zhang, Xudong Zhu, Xingming Zhang, Jindong Dai, Pengfei Shen, Hao Zeng

**Affiliations:** Department of Urology, Institute of Urology, West China Hospital, Sichuan University, Chengdu, China

**Keywords:** clinical management, prostate cancer, radiotherapy, surgery, SEER

## Abstract

**Purpose:**

We aimed to compare the efficacy of radical prostatectomy (RP) + extended pelvic lymph node dissection (ePLND) and radiotherapy (RT) in localized prostate cancer (PCa) patients with a risk of lymph node invasion (LNI) over 5%.

**Methods:**

The Surveillance, Epidemiology, and End Results (SEER) databases were used to identify patients with PCa from 2010 to 2014. Propensity score matching (PSM) was performed to balance baseline characteristics between patients in different treatment groups. Kaplan-Meier curves and Cox regression were used to assess the effects of treatments on cancer-specific survival (CSS) and overall survival (OS).

**Results:**

Overall 20584 patients were included in this study, with 4,057 and 16,527 patients receiving RP + ePLND and RT, respectively. After PSM, patients with RP + ePLND had similar CSS (5-year CSS rate: 97.8% vs. 97.2%, P=0.310) but longer OS (5-year OS rate: 96.0% vs. 90.8%, P<0.001) compared to those receiving RT. When separating RT cohort into external beam radiotherapy (EBRT) group and EBRT+ brachytherapy (BT) group, treatments with RP + ePLND and EBRT+ BT achieved equivalent OS and were both superior to EBRT alone (5-year OS rate: 96.0% vs. 94.4% vs. 90.0%, P<0.001). Subgroup analyses and multivariate analyses further confirmed the superiority of RP + ePLND and EBRT+ BT.

**Conclusion:**

RP + ePLND and EBRT + BT were associated with better survival outcomes compared to EBRT alone in PCa patients with a probability of LNI over 5%. However, no survival difference was observed between RP + ePLND and EBRT + BT.

## Introduction

Prostate cancer (PCa) is the most common malignancy in men in the United States, with 191,930 estimated new cases and 33,330 estimated deaths in 2020 (1). Although an increasing number of patients are diagnosed as PCa at early stages with the introduction of prostate-specific antigen (PSA) screening, approximately 12% of patients still have lymph node invasion (LNI) at initial presentation (1). It has been widely reported that the presence of positive lymph nodes represents an adverse pathologic finding associated with poor survival outcomes in patients with PCa (2–4). At present, extended pelvic lymph node dissection (ePLND) represents the most accurate procedure for nodal staging and several pre-operative tools have been established to predict the individual risk of LNI in patients with PCa and select candidates for ePLND (5–7). Given the essential role of ePLND in tumor staging and its potential therapeutic effect, the current European Association of Urology (EAU) guidelines recommend performing radical prostatectomy (RP) and ePLND for patients with a risk of LNI greater than 5% according to Briganti nomogram (8). Meanwhile, radiotherapy (RT) with prophylactic nodal radiation is also the standard of care for clinically localized PCa, especially in unfavorable intermediate-risk and high-risk patients (8, 9). However, there is uncertainty regarding the efficacy of RP plus ePLND compared to RT in localized PCa patients with a relatively high risk of LNI.

Against this backdrop, this study aimed to use the Surveillance, Epidemiology, and End Results (SEER) database to examine the effectiveness of RP plus ePLND versus RT in treating localized PCa patients with a probability of LNI over 5%.

## Patients and Methods

### Study Population and Data

This study was approval by institutional ethics committee and informed consents were waived due to the de-identified nature of SEER database. Data were extracted from the SEER database using SEER*Stat, version 8.3.6. Patients with histologically confirmed adenocarcinoma of the prostate [International Classification of Disease for Oncology (61.9); histological code: 8140] diagnosed from 2010 to 2014 were identified. Inclusion criteria were: 1) patients with complete baseline clinicopathologic data; 2) patients with a probability of LNI>5% based on 2012-Briganti nomogram; 3) patients received RP plus ePLND or RT as primary treatment. Patients with a tumor stage>T3, clinically positive nodes, distant metastasis, neoadjuvant therapies, other malignancies, or missing follow-up data were excluded from this study.

Data regarding the age of diagnosis, race, clinical T (cT) stage, PSA value, Gleason score (GS) at biopsy, number of positive or negative cores, number of lymph nodes positive, number of lymph nodes examined, survival time and cause of death were collected for each patient. EPLND was defined as dissected lymph nodes ≥10 according to previous reports (5–7).

### Endpoints and Statistical Analysis

Primary endpoints were cancer-specific survival (CSS) and overall survival (OS), defined by the SEER database. The probability of LNI was calculated based on the 2012-Briganti nomogram. Continuous variables were presented using the median and interquartile range (IQR). Frequency and proportion were reported for categorical variables. Chi-square test and Student’s *t* test were used to compare categorical and continuous variables of baseline characteristics, respectively. To balance the baseline clinicopathologic characteristics of two treatment groups, 1:1 propensity score matching (PSM) was conducted with a caliper of 0.001. Survival estimates were generated and compared using the Kaplan-Meier method and log-rank test, respectively. To further assess the survival differences between treatment modalities, patients with RT were subdivided into external beam radiotherapy (EBRT) group and EBRT+ brachytherapy (BT) group. Besides, subgroup analyses were conducted according to baseline features and presented with forest plots. At last, univariate and multivariate analyses were performed using Cox proportional hazards regressions to determine independent predictors of survival. SPSS, version 25.0 was used for all statistical analyses. A two-sided P value<0.05 indicated statistical significance.

## Results

### Patients Characteristics

Overall, 20,584 patients with a risk of LNI>5% were identified from the SEER database between 2010 and 2014. Among them, 4,057 and 16,527 patients received RP + ePLND and RT, respectively. In the RT group, 14,134 and 2,393 patients were treated with EBRT and EBRT + BT, respectively. In the RP + ePLND group, a median of 14 lymph nodes (IQR 12–19) were removed and 714 patients (17.6%) harbored LNI. A total of 549 (13.5%) patients received adjuvant or salvage RT after surgery. As shown in **Table 1**, compared to patients treated with RT, patients receiving RP + ePLND were younger and had significantly lower PSA value, lower probability of LNI but higher cT stage. After PSM, the baseline clinicopathologic characteristics were well balanced between the RP + ePLND group and RT group.

**Table 1:** Baseline characteristics of patients treated with RP + ePLND and RT in overall cohort and propensity-score matched cohort.

Characteristics	Overall cohort			Propensity-score matched cohort		
	RP + ePLND(n=4057)	RT(n=16527)	P value	RP + ePLND(n=3675)	RT(n=3675)	P value
Age, years, n (%)						
Median (IQR)	62 (56–66)	68 (62–74)		62 (56–66)	64 (60–67)	
<70	3567 (87.9%)	8817 (53.3%)	<0.001	3232 (87.9%)	3218 (87.6%)	0.618
≥70	490 (12.1%)	7710 (46.7%)		443 (12.1%)	457 (12.4%)	
Race, n (%)						
Black	509 (12.5%)	3310 (20.0%)	<0.001	491 (13.4%)	517 (14.1%)	0.099
White	3256 (80.3%)	11826 (71.6%)		2981 (81.1%)	2917 (79.4%)	
Others	292 (7.2%)	1391 (8.4%)		203 (5.5%)	241 (6.5%)	
D’Amico risk stratification, n (%)						
Low	81 (2.0%)	413 (2.5%)	0.073	74 (2.0%)	56 (1.5%)	0.233
Intermediate	1456 (35.9%)	6082 (36.8%)		1328 (36.1%)	1360 (37.0%)	
High	2520 (62.1%)	10032 (60.7%)		2273 (61.9%)	2259 (61.5%)	
Clinical T stage, n (%)						
T1	2018 (49.7%)	8754 (53.0%)	<0.001	1985 (54.0%)	1990 (54.1%)	0.295
T2	1696 (41.8%)	6694 (40.5%)		1361 (37.0%)	1392 (37.9%)	
T3	343 (8.5%)	1079 (6.5%)		329 (9.0%)	293 (8.0%)	
PSA, ng/ml, n (%)						
Median (IQR)	7.6 (5.3–13.1)	8.8 (6.0–16.1)		7.8 (5.4–13.6)	8.4 (5.7–13.9)	
≤20	3500 (86.3%)	13346 (80.8%)	<0.001	3121 (84.9%)	3148 (85.7%)	0.374
>20	557 (13.7%)	3181 (19.2%)		554 (15.1%)	527 (14.3%)	
Gleason score at biopsy, n (%)						
≤7	2382 (58.7%)	9568 (57.9%)	0.343	2027 (55.2%)	2029 (55.2%)	0.963
≥8	1675 (41.3%)	6959 (42.1%)		1648 (44.8%)	1646 (44.8%)	
Probability of LNI, n (%)						
≤20%	2935 (72.3%)	11400 (69.0%)	<0.001	2601 (70.8%)	2631 (71.6%)	0.440
>20%	1122 (27.7%)	5127 (31.0%)		1074 (29.2%)	1044 (28.4%)	
Number of removed nodes, median (IQR)						
	14 (12–19)			15 (11–20)		
LNI, n (%)						
	714 (17.6)			662 (18.0)		
Adjuvant/salvage RT, n (%)						
	549 (13.5%)			513 (14.0%)		

RP, radical prostatectomy; ePLND, extended pelvic lymph node dissection; RT, radiotherapy; IQR interquartile range; PSA, prostate-specific antigen; LNI, Lymph node invasion.

### Treatment Effect of RP + ePLND Versus RT

**Figure 1:**
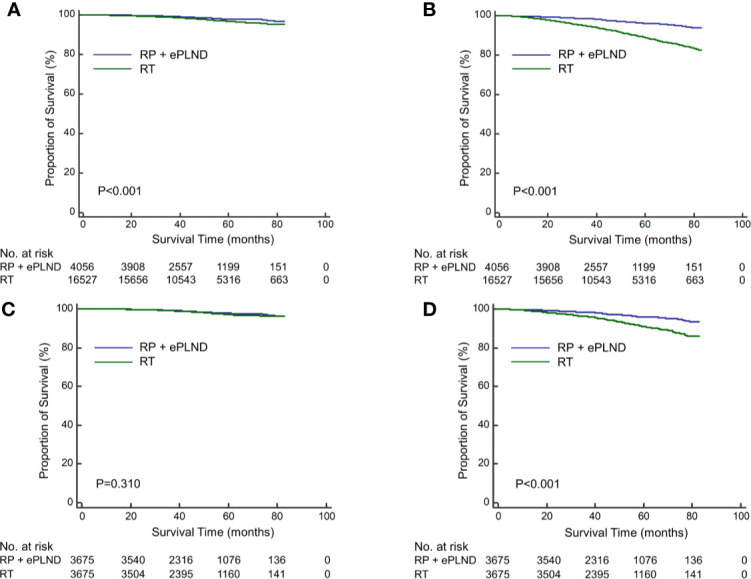
Kaplan-Meier curves of cancer-specific survival **(A)** and overall survival **(B)** for patients receiving RP + ePLND and RT in the entire cohort before PSM; Kaplan-Meier curves of cancer-specific survival **(C)** and overall survival **(D)** for patients receiving RP + ePLND and RT after PSM.

The median follow-up was 50 months for overall patients, 48 and 52 months for patients with RP + ePLND and RT, respectively. As shown in **Figures 1A, B**, compare to patients with RT, those receiving RP + ePLND were associated with significantly improved CSS (5-year CSS rate: 98.0% vs. 96.6%, P<0.001) and OS (5-year OS rate: 96.1% vs. 88.7%, P<0.001) before PSM. After PSM, the CSS was comparable between two treatment groups (5-year CSS rate: 97.8% vs. 97.2%, P=0.310), while patients treated with RP + ePLND had longer OS (5-year OS rate: 96.0% vs. 90.8%, P<0.001) than patients with RT (**Figures 1C, D**).

### Treatment Effect of RP + ePLND Versus EBRT Versus EBRT + BT

**Figure 2:**
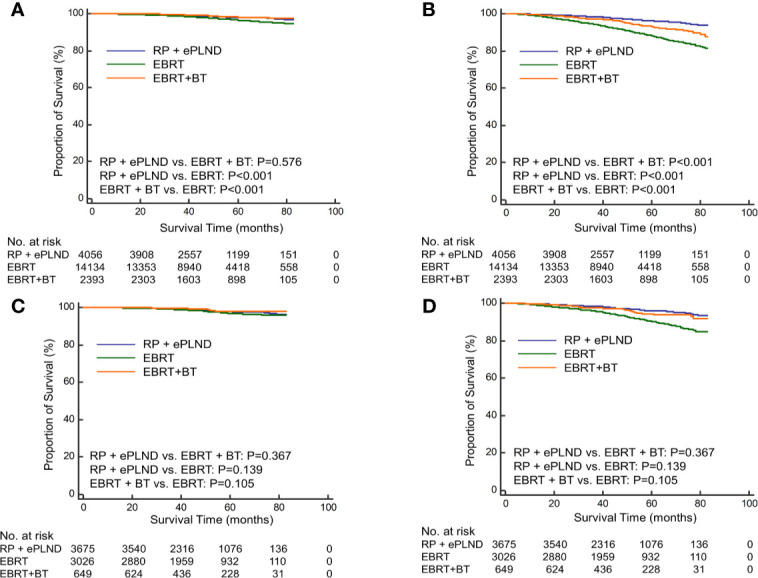
Kaplan-Meier curves of cancer-specific survival **(A)** and overall survival **(B)** for patients receiving RP + ePLND, EBRT alone and EBRT + BT in the entire cohort before PSM; Kaplan-Meier curves of cancer-specific survival **(C)** and overall survival **(D)** for patients receiving RP + ePLND, EBRT alone and EBRT + BT in after PSM.

To further investigate the efficacy of different radiation modalities, the RT cohort was subdivided into EBRT group and EBRT + BT group. Before PSM, patients underwent RP + ePLND had similar CSS (5-year CSS rate: 98.0% vs. 98.0%, P=0.569) with those receiving EBRT + BT but longer CSS (5-year CSS rate: 98.0% vs. 96.3%, P<0.001) compared to men with EBRT alone (**Figure 2A**). In terms of OS, RP + ePLND had obvious superiority to EBRT + BT or EBRT alone (5-year OS rate: 96.1% vs. 93.1% vs. 87.9%, P<0.001, **Figure 2B**). After PSM, as shown in **Figure 2C**, CSS was comparable among three treatment groups with a 5-year CSS was 97.8%, 98.0%, and 97.0% (P=0.136) for patients with RP + ePLND, EBRT + BT and EBRT alone, respectively. However, patients receiving RP + ePLND or EBRT + BT had significantly longer OS than those receiving EBRT alone (5-year OS rate: 96.0% vs. 94.4% vs. 90.0%, P<0.001, **Figure 2D**). When comparing the efficacy of RP + ePLND versus EBRT + BT, no statistical difference was observed for OS(P=0.167).

### Subgroup Analyses and Multivariate Cox Regression

**Figure 3:**
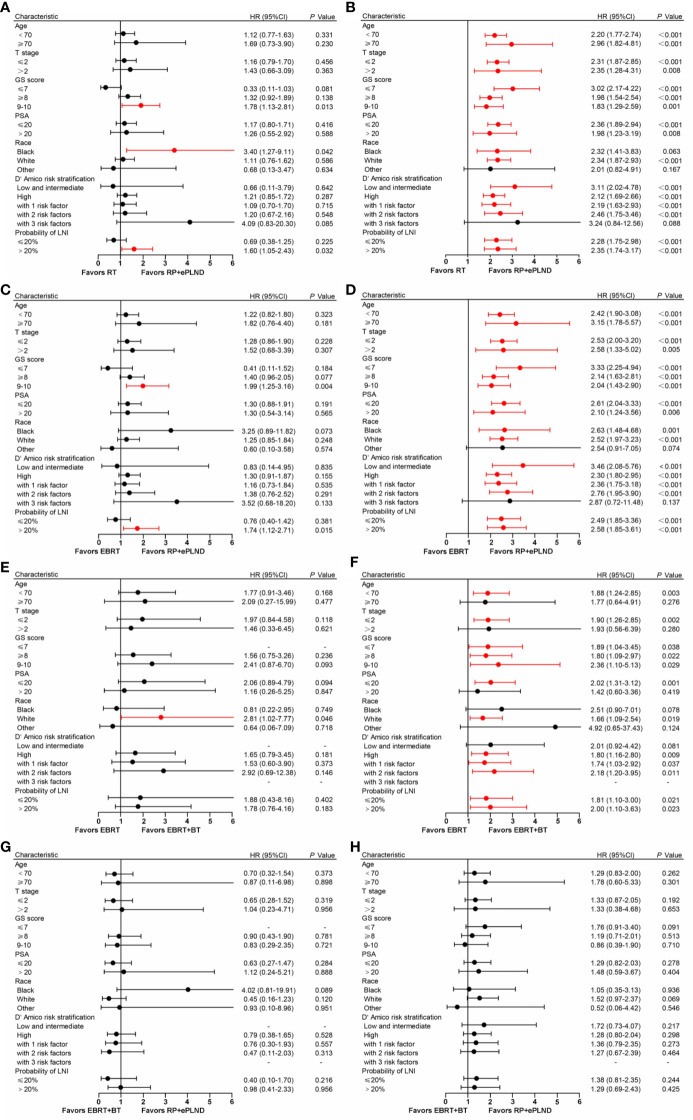
Forest plot showing the effect of RP + ePLND versus RT on cancer-specific survival **(A)** and overall survival **(B)** in patients with different baseline characteristics; Forest plot showing the effect of RP + ePLND versus EBRT alone on cancer-specific survival **(C)** and overall survival **(D)** in patients with different baseline characteristics; Forest plot showing the effect of EBRT + BT versus EBRT alone on cancer-specific survival **(E)** and overall survival **(F)** in patients with different baseline characteristics; Forest plot showing the effect of RP + ePLND versus EBRT + BT on cancer-specific survival **(G)** and overall survival **(H)** in patients with different baseline characteristics.

To select the optimal treatment for patients with distinct features, subgroup analyses were conducted based on baseline clinicopathologic characteristics in post-PSM cohorts. As shown in **Figure 3**, the results were basically consistent with prior analyses that patients in different treatment groups had similar CSS, while men receiving RP + ePLND or EBRT + BT could gain more OS benefits than those with EBRT alone. To note, patients with a higher risk of LNI (>20%) or a GS 9-10 might have not only longer CSS but also improved OS when treated with RP + ePLND.

After adjusting for clinicopathologic covariates, multivariate Cox regression analyses indicated that neither RP + ePLND nor RT was an independent predictor of CSS. However, RP + ePLND/EBRT + BT along with the lower probability of LNI, lower GS, younger age, and low/intermediate-risk disease independently predicted better OS. No survival difference was observed between RP + ePLND group and EBRT + BT group in multivariate analyses (**Table 2**).

**Table 2:** Univariate and Multivariate analyses of cancer-specific survival and overall survival for patients in post-propensity score matching cohort.

	Cancer-specific survival				Overall survival			
	Univariate analyses		Multivariate analyses		Univariate analyses		Multivariate analyses	
	HR (96% CI)	p value	HR (96% CI)	p value	HR (96% CI)	p value	HR (96% CI)	p value
Age (years)								
≥70 vs. <70	1.46 (0.92–2.31)	0.106			1.45 (1.11–1.89)	0.006	1.33 (1.02–1.74)	0.038
Race								
White vs. Black	1.20 (0.71–2.02)	0.503			0.87 (0.66–1.14)	0.320		
Other vs. Black	0.79 (0.31–2.01)	0.614	^*^		0.68 (0.41–1.13)	0.136		
Treatment								
RT vs. RP + ePLND	1.20 (0.85–1.69)	0.310			2.31 (1.85–2.87)	<0.001	2.29 (1.84–2.85)	<0.001
EBRT vs. RP + ePLND	1.31 (0.92–1.87)	0.139			2.53 (2.03–3.16)	<0.001	2.48 (1.99–3.10)	<0.001
EBRT + BT vs. RP + ePLND	0.71 (0.34–1.49)	0.367			1.33 (0.89–2.00)	0.170	1.40 (0.93–2.10)	0.110
EBRT vs. EBRT + BT	1.84 (0.88–3.83)	0.105			1.90 (1.29–2.80)	0.001	1.78 (1.21–2.62)	0.003
Probability of LNI*	1.02 (1.01–1.02)	<0.001	1.01 (1.01–1.02)	<0.001	1.01 (1.00–1.01)	<0.001	1.01 (1.00–1.01)	<0.001
D’Amico risk stratification								
High vs. Low/Intermediate	15.88 (6.50–38.82)	<0.001	8.98 (3.60–22.39)	<0.001^#^	2.31 (1.81–2.94)	<0.001	1.76 (1.35–2.29)	<0.001^
PSA, ng/ml								
>20 vs. ≤20	1.23 (0.78–1.94)	0.384			1.29 (0.99–1.69)	0.057	0.93 (0.68–1.27)	0.638
Clinical T stage								
>T2 vs. ≤T2	2.76 (1.79–4.24)	<0.001	0.86 (0.50–1.47)	0.571	1.27 (1.10–1.47)	0.001	0.93 (0.77–1.13)	0.471
Gleason score								
≥8 vs. ≤7	12.85 (7.09–23.26)	<0.001	8.97 (4.90–16.40)	<0.001	2.32 (1.88–2.86)	<0.001	1.87 (1.50–2.34)	<0.001

HR, hazard ratio; RP, radical prostatectomy; ePLND, extended pelvic lymph node dissection; RT, radiotherapy; EBRT, external beam radiotherapy; BT, brachytherapy; PSA, prostate-specific antigen; LNI, Lymph node invasion; *As continuous variate, ^#^Adjusted for: probability of LNI, ^Adjusted for: age, treatment, and probability of LNI.

## Discussion

Considering the poor prognosis associated with LNI, much effort has been invested into predicting the risk of LNI in patients with PCa and seeking subsequent therapies for men with LNI (5–7). Among various prediction tools, the 2012-Briganti nomogram has been validated in North American patients and was reported to have high accuracy and best performance characteristics in the decision curve analysis (10). Despite that the 2012-Briganti nomogram has been updated recently with inclusion of MRI features and improved predictive power, the new nomograms have not been widely externally validated (11, 12). Thus, current international guidelines still recommend to select patients for RP + ePLND with a cut-off point of 5% based on 2012-Briganti nomogram (8). At the same time, RT is also the standard treatment for localized PCa regardless of the risk of LNI (8, 9). Although a large number of studies have investigated the optimal therapy for overall patients with localized PCa, men with a relatively high risk of LNI stills represent a grey zone as no comparison has been made of currently available treatments in this setting (13–20). To the best of our knowledge, this was the first study to compare the efficacy of RP + ePLND and RT in localized PCa patients with a relatively high risk of LNI.

In this study, we observed that patients receiving RP + ePLND were younger and associated with lower PSA value, lower risk of LNI. Consistently, previous population-based studies also reported that compared to men with RT, those who underwent RP had more favorable clinicopathologic characteristics (15, 18). Thus, it was not surprising that RP + ePLND achieved improved survival outcomes than RT in overall patients. Interestingly, after adjusting clinicopathologic features, patients with RP + ePLND still had longer survival than those with RT. When comparing men who underwent different types of RT versus RP + ePLND, RP + ePLND, and EBRT + BT showed significant superiority to EBRT alone. However, no survival difference was observed between patients with RP + ePLND and EBRT + BT.

In line with our results, several previous studies demonstrated that RP provided more survival benefits than RT in patients with high-risk PCa who were more likely to harbor positive nodes (14, 16, 17, 20). In theory, RP + ePLND could remove the primary prostate lesion as well as potential micro-metastases in pelvic lymph nodes, resulting in long-term disease control. Meanwhile, RT with concomitant prophylactic nodal radiation could also achieve satisfied survival outcomes by eliminating local cancer cells. Nevertheless, the therapeutic role of ePLND in addition to RP remained controversial, it provided important nodal information and aided adjuvant treatments in selected patients (21–23). On the contrary, the efficacy of RT might be highly limited by the absence of accurate pathological nodal staging and corresponding adjuvant therapies, which finally led to undertreatment in patients in our study who were at a relatively higher risk of LNI. This point might partly explain the better survival benefit of RP + ePLND than RT in this setting.

Currently, RT options for PCa include EBRT alone and EBRT + BT, with the latter constituting extreme dose escalation. Several randomized trials suggested a biochemical recurrence-free survival benefit to EBRT+BT over EBRT but failed to show significant improvements in long-term survival outcomes (24–26). Therefore, the question arises simultaneously whether RP + ePLND and different RT modalities offer varied survival benefits in PCa patients with a probability of LNI over 5%. In this study, we found that RP + ePLND and EBRT + BT achieved similar OS, and both of them outperformed EBRT alone. This finding was consistent with the study by Ennis and colleagues, which indicated that, after adjusting lymph node status, there was no statistical difference in survival between RP and EBRT + BT (15). However, EBRT plus androgen deprivation therapy (ADT) was associated with higher mortality than RP. The superior results observed in our study with the combination of EBRT and BT might be due to an increased dose of radiation to the prostate and periprostatic area as well as radiobiological factors associated with shorter duration (26). Thus, our findings further confirmed the perspective that dose escalation in a short delivery time should be the key to improve the effectiveness of RT in PCa (27, 28).

To further select patients for preferred treatment, we conducted subgroup analyses based on baseline characteristics. We found that men with a higher risk of LNI or a GS≥9 could gain more CSS and OS benefit form RP + ePLND and EBRT + BT, which suggested that more intense treatments were needed in this setting. In line with our results, another recent study has also indicated the treatment with Max RP (RP with adjuvant therapies) or MaxRT (EBRT + BT + ADT) for PCa patients with a GS≥9 could lead to equivalent and favorable CSS and OS outcomes (19). Intriguingly, it seemed that race may also play a role in the response to different therapies as no survival difference was seen between treatment groups in men of other races such as American Indians and Asians. Further studies are warranted to verify our results and novel stratification tools are needed to select candidates for optimal therapies.

Our findings should be interpreted with caution as this study is not devoid of limitations. First, the present results were derived from retrospective analyses with inherent bias, whereas we used the PSM method to balance the differential clinicopathologic features between groups, which helped minimize the impact of potential bias. Second, the definition of ePLND was based on the number of dissected nodes due to the lack of anatomic nodal information. However, a median number of 14 removed nodes was achieved in our study, which was equivalent to that in previous reports based on anatomical ePLND (5–7). Third, due to the limitation of SEER databases, the protocols of RT and data regarding the use of ADT were not available. As such, the effectiveness of RT might be underestimated. At last, with a median follow-up of 50 months, the data concerning CSS might not be mature enough to address significant differences between treatments. Large prospective trials with complete treatment details are needed to validate our findings.

## Conclusion

Our study is the first to compare the efficacy of RP + ePLND and RT in PCa patients with a relatively high risk of LNI. We demonstrated that RP + ePLND and EBRT + BT were associated with better survival outcomes compared to EBRT alone in men with a probability of LNI over 5%. However, no significant survival difference was observed between RP + ePLND and EBRT + BT.

## Data Availability Statement

Publicly available datasets were analyzed in this study. This data can be found here: Surveillance, Epidemiology, and End Results (SEER) database (https://seer.cancer.gov/).

## Ethics Statement

The studies involving human participants were reviewed and approved by The ethics committee of West China Hospital, Sichuan University. Written informed consent for participation was not required for this study in accordance with the national legislation and the institutional requirements.

## Author Contributions

JC: Conceptualization, software, data curation, formal analysis, data interpretation, manuscript drafting, and manuscript editing. YN: Conceptualization, data curation, formal analysis, data interpretation, manuscript drafting, and manuscript editing. GS, SZ, JZ, ZW, HRZ, XDZ, XMZ, and JD: Data curation, data interpretation, and manuscript editing. PS, and HZ: Conceptualization, formal analysis, data curation, data interpretation, and manuscript editing. All authors contributed to the article and approved the submitted version.

## Funding

This work was supported by the National Natural Science Foundation of China (NSFC 81672547 and 81974398); The 1.3.5 project for disciplines of excellence, West China Hospital, Sichuan University.

## Conflict of Interest

The authors declare that the research was conducted in the absence of any commercial or financial relationships that could be construed as a potential conflict of interest.
